# Increased IL-21 secretion by aged CD4+T cells is associated with prolonged STAT-4 activation and CMV seropositivity

**DOI:** 10.18632/aging.100490

**Published:** 2012-09-29

**Authors:** Anshu Agrawal, Houfen Su, Justine Chen, Kathryn Osann, Sudhanshu Agrawal, Sudhir Gupta

**Affiliations:** Department of Medicine, Division of Basic and Clinical Immunology, University of California, Irvine, CA 92617, USA

**Keywords:** Aging, CD4+ T cells, IL-21, T follicular helper cells, STAT-4, Cytomegalovirus

## Abstract

Advancing age leads to significant decline in immune functions. IL-21 is produced primarily by T follicular helper (Tfh) cells and is required for effective immune cell functions. Here we compared the induction of IL-21 in aged and young subjects. Our investigation demonstrates that CD4+T cells from healthy elderly individuals (age ≥ 65) secreted significantly higher levels of IL-21 on priming with aged and young dendritic cells (DC). Though the aged and young DCs secreted comparable levels of IL-12 on stimulation with anti-CD40 antibody and LPS, culture of DCs with aged CD4+ T cells resulted in increased production of IL-21 as compared to that with young CD4+ T cells. Further examination revealed that the response of aged naïve CD4+ T cells to IL-12 was altered, resulting in increased differentiation of aged Th cells towards Tfh cells. Investigation into the signaling mechanism suggested that phosphorylation of STAT-4 in response to IL-12 was sustained for a longer duration in aged CD4+ T cells as compared to CD4+ T cells from young subjects. Additional analysis demonstrated that increased IL-21 secretion correlated with chronic CMV infection in aged subjects. These findings indicate that chronic CMV infection alters the response of aged CD4+ T cells to IL-12 resulting in an increased secretion of IL-21 and that aging affects Tfh cell responses in humans which may contribute to age-associated inflammation and immune dysfunctions.

## INTRODUCTION

The immune system undergoes significant changes with advancing age [1, 2]. The functions of both innate and adaptive immune cells are significantly impacted with age resulting in increased susceptibility to infections as well as reduced response to vaccination [1-5]. For example, DCs from aged are impaired in their response to infections but display increased reactivity to self antigens which results in low grade chronic inflammation and autoimmunity [6, 7]. T and B cell functions are similarly affected. Thymic involution leads to a decrease in naïve T cell population which is accompanied by accumulation of dysfunctional memory T cells [2, 8]. The magnitude and quality of B cell responses are also compromised [9-11]. However, the mechanisms underlying the age-associated immune dysfunctions are not well understood.

Studies in the past few years have led to the identification of a novel subset of Th cells known as the T follicular helper cells (Tfh cells). It has been demonstrated that IL-12 production by activated dendritic cells (DCs) induces naïve CD4+ T cells to polarize to IL-21-producing T follicular helper (Tfh)-like cells [12, 13]. IL-21 is a recently discovered member of the type I cytokine family, which includes IL-2 and IL-15 [14]. Similar to other members of the cytokine family, IL-21 also signals through the common γ-chain and a unique IL-21 receptor (IL-21R) [15]. IL-21R is widely expressed in cells lymphoid-lineage, and regulates the proliferation and differentiation of the T and B lymphocytes [14]. Recent studies suggest that IL-21 is a key component of CD4 T cell help that is required for maintaining the CD8+ T cell responses and inducing B cell antibody response. In particular, IL-21 stimulates B cell proliferation, promotes B cell maturation and IgG production including the generation of long-lived and high affinity plasma cells and memory cells that are crucial for long-term protection against infections. [16,17]. Furthermore, IL-21 promotes the development of Th17 and Tfh cells, modulates the cytotoxic activity and survival of NK and CD8+ T cells, and suppresses the maturation of DCs. It is also implicated in the development of autoimmune disease and has antitumor activity [18, 19].

Given the importance of IL-21 in regulating immune functions we investigated whether IL-21 production is altered with age in humans.

## RESULTS

### Increased IL-21 secretion from aged subjects is not due to age-associated alteration in dendritic cell functions

**Figure 1:**
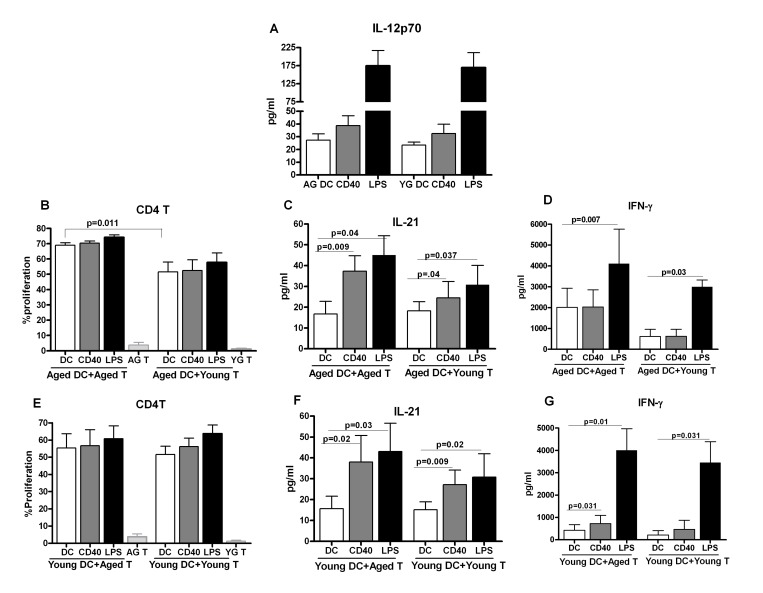
Increased IL-21 secretion from aged subjects is not due to age-associated alteration in dendritic cell function **A.** Bar graph depicts the levels of IL-12p70 in the supernatant from stimulated aged and young DC. **B.** Bar graph depicts the percent proliferation of aged and young CD4+ T cells after culture with aged DCs. **C.** Bar graph depicts the level of IL-21 in the supernatant of aged and young CD4+ T cells after culture with aged DCs. **D.** Bar graph depicts the level of IFN-g in the supernatant of aged and young CD4+ T cells after culture with aged DCs. **E.** Bar graph depicts the percent proliferation of aged and young CD4+ T cells after culture with young DCs. **F.** Bar graph depicts the level of IL-21 in the supernatant of aged and young CD4+ T cells after culture with young DCs. **G.** Bar graph depicts the level of IFN-γ in the supernatant of aged and young CD4+ T cells after culture with young DCs. Data is mean +/− S.E. of 8 different aged and young subjects.

It has been recently reported that IL-12 secreted by DCs acts on CD4+ T cells to induce IL-21 producing Tfh cells in humans [12]. IL-21 enhances germinal center formation and B cell differentiation to plasma cells as well as augments the cytotoxic activity of CD8+ T cells [16, 17]. Since advancing age results in substantial decrease the above immune functions, we investigated whether the capacity of DCs to prime IL-21 producing Tfh cells is altered with age. To determine this, first we compared the production of IL-12 between DCs from aged and young subjects following stimulation with anti-CD40 antibody and LPS based on Schimdt et al [12]. As is evident from Figure 1A, IL-12 secretion by DCs was comparable between aged and young subjects.

Next, we determined the capacity of anti-CD40 and LPS- primed aged and young DCs to polarize Th cells. Aged DCs unstimulated and stimulated with anti-CD40 and LPS were cultured with allogeniec young and aged CD4+ T cells for six days to determine proliferation and cytokine secretion. Basal level of proliferation was significantly higher (p<0.05) in unstimulated aged DC-aged T cell co-culture cells as compared to unstimulated aged DC-young T cell co-culture (Figure 1B). Co-culture of anti-CD40 and LPS-stimulated aged DCs with aged T cells was also enhanced compared to aged DC-young T cell co-culture though the increase compared to unstimulated DC condition was comparable. Determination of cytokines in the supernatant by ELISA revealed that co-culture of aged DCs with young T cells resulted in increased secretion of IL-21 in anti CD40 and LPS stimulated groups (Figure 1C) suggesting that the capacity of aged DCs to induce IL-21 is not affected with age. IFN-γ showed a significant increase only in the LPS stimulated group (Figure 1D). Remarkably, co-culture of aged DCs with allogeneic aged T cells resulted in significantly enhanced (p<0.05) secretion of IL-21 from anti-CD40 and LPS-stimulated groups compared to co-culture of aged DCs with young T cells (Figure 1C). IFN-γ secretion was significantly higher (p<0.05) at baseline and after stimulation with LPS in aged DC and aged T cell co-culture compared to aged DC and young T cell co-culture (Figure 1D). However, the increase in IFN-γ in the stimulated DC groups relative to unstimulated DC condition was comparable between aged DC-aged T and aged DC-young T co-culture. IL-17 and IL-10 were below the detection limits. These data suggest that IL-21 secretion is increased in aged DC-aged T cell co culture compared to aged DC, young T cell co-culture. This could be due to an increased capacity of aged DC to induce IL-21 from T cells or due to age-associated increase in the capacity aged T cells to secrete IL-21.

To determine whether the increased IL-21 secretion observed in aged DC, aged T co-cultures is due to age-associated modifications in DCs or T cells, the experiments were performed with young DCs (to rule out DC defect) instead of aged DCs. The proliferation was comparable between young DC-aged T cells and young DC-young T cells co-culture in both unstimulated and anti-CD40 and LPS-stimulated groups (Figure 1E). Despite comparable proliferation, significantly increased (p<0.05) IL-21 secretion was observed in young DC-aged T cells co-culture compared to young DC-young T cells co-culture suggesting that aged T cells have enhanced capacity to produce IL-21(Figure 1F). IFN-γ secretion was not significantly different between the two groups (Figure 1G). IL-17 and IL-10 were below the detection limits.

In summary, the above data suggest that aged T cells display an increased capacity to produce IL-21 which is not a consequence of age-associated modifications in either IL-12 production or T cell priming function of aged DCs.

### Intrinsic age-associated changes in CD4+ T cells from aging subjects are responsible for the increased in IL-21 secretion

**Figure 2:**
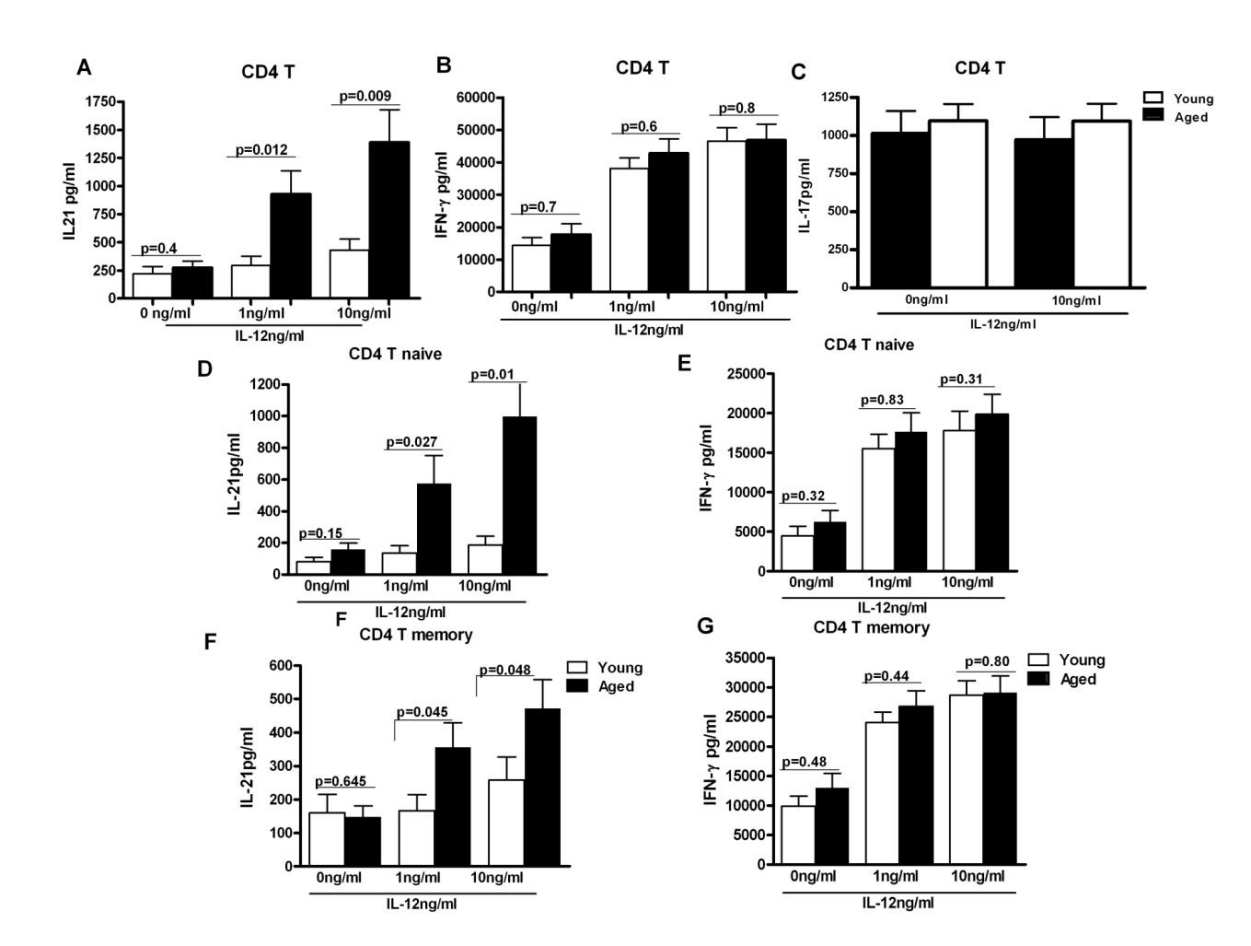
IL-21 secretion is increased from CD4+ T cells from aged subjects **A, B, C** Bar graph depicts the levels of IL-21 (**A**), IFN-γ (**B**), IL-17 (**C**) in the supernatant from aged and young total CD4+ T cells after stimulation with IL-12 for 5 days. Data is mean +/− S.E. of 23 different aged and young subjects. **D, F.** Bar graph depicts the levels of IL-21 secreted from aged and young, Naïve CD4+ T cells (**D**) and memory CD4+ T cells (**F**) after stimulation with IL-12 for 5 days. **E, G.** Bar graph depicts the levels of IL-21 secreted from aged and young, Naïve CD4+ T cells (**E**) and memory CD4+ T cells (G) after stimulation with IL-12 for 5 days. Data is mean +/− S.E. of 15 different aged and young subjects.

To confirm r our observations in Figure 1 and to rule out the effect of other DC cytokines and factors, we exposed purified aged and young CD4+ T cells to similar levels of recombinant IL-12 for 5 days. Supernatants were collected and assayed for IL-21 and IFN-γ secretion by specific ELISA. As shown in Figure 2A, CD4+ T cells from aged subjects, in a concentration dependent manner, secreted significantly increased (p<0.05) levels of IL-21. Though the increase was observed in both naïve (CD4+, CD45RA+) and memory (CD4+, CD45RO+) T cell subsets, naïve T cells secreted significantly (p<0.05) larger quantities of IL-21 as compared to memory T cells (Figure 2D &E). The results were further confirmed with intracellular staining (supplementary Figure 1) where we also observed that majority of IL-21 secretion was from CD4+, CD45RA+, CCR7+ naïve T cells. Similar increase in IL-21 secretion from naïve CD4+T cells as compared to memory CD4+T cells has been reported [13]. The secretion of IFN-γ was comparable between aged and young subjects (Figure2B, F & G). IL-17 secretion was also comparable in aged and young subjects (Figure 2C). No IL-21 secretion was observed from purified CD8+ T cells (unpublished observations). These data demonstrate that the response of aged CD4+ T cells to IL-12 is altered resulting in enhanced secretion of IL-21.

In the aged subjects there were several subgroups based on comorbidities (Table 1). For some of the subgroups we had enough subjects to do a subgroup analysis. Osteoarthritis was the most common comorbid condition in the aged population studied. IL-21 levels were comparable between arthritis positive and negative subjects (p=0.46). Aged subjects with hypertension and dyslipidemia were also similar in their induction of IL-21 (p>0.4). Many of the aged subjects were also taking vitamins and antioxidants however, we did not observe any difference in IL-21 levels between the two groups (p>0.1). Furthermore, subjects were off antioxidants for at least one week prior to blood draw. Based on these subgroup analyses, it is apparent that these comorbid factors did not influence IL-21 production by aged CD4+ T cells.

**Table 1:** Description of the aged and young cohorts

	Young n=23	Aged n=23
Age (range)	25 (21-35)	80 (65-88)
Gender, female	13 (56%)	15 (62%)
**Comorbidities**		
Osteoarthritis	0	11 (48%)
Hypertension	0	9(39%)
Dyslipidemia	0	6 (26%)
Diabetes	0	0(0%)
**Medications**		
Vitamins	0	17 (74%)
Antioxidants	0	12 (52%)

### Increased IL-21 secretion in CD4+ T cells from aged subjects is not a consequence of increased IL-12 receptor expression

**Figure 3:**
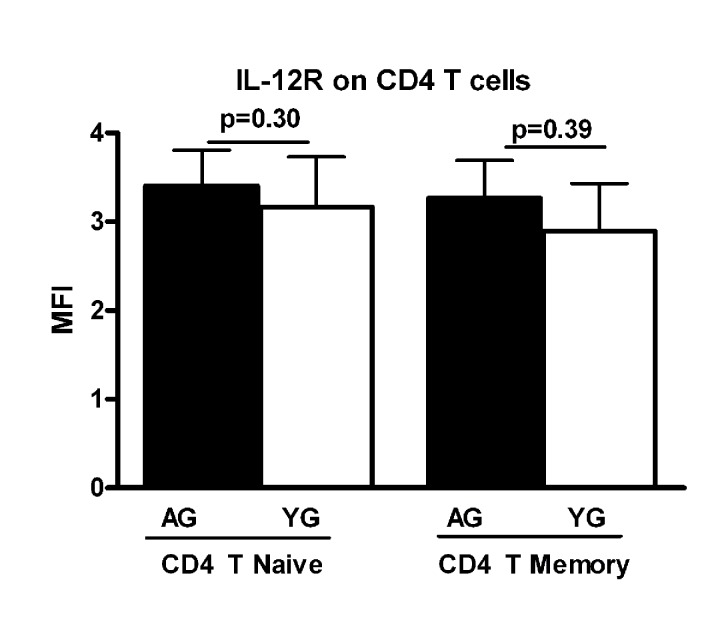
Expression of IL-12R is comparable on aged and young subjects Bar graph depicts the mean fluorescence intensity (MFI) of IL-12 receptor on naïve and memory CD4+ T cells from aged and young subjects. Data is mean +/− S.E. of 15 different aged and young subjects.

Next we investigated if the expression of IL-12 receptor is increased in aged CD4+ T cells. PBMCs from aged and young subjects were surface stained for CD4+, CD45RA and IL-12R using specific antibodies (BD Bioscience, RnD Systems) and isotype controls. Cells were acquired using BD FACScaliber. Gated CD4+ T naïve (CD4+, CD45RA+) and memory (CD4+, CD45RA-) cells were analyzed for IL-12R expression using Flow jo software. The expression of IL-12R was comparable in both naïve and memory CD4+ T cells from aged and young subjects (Figure 3). This suggests that the increased IL-21 secretion during aging is not due to an enhanced expression of IL-12R on CD4+ T cells. This observation is further supported by the fact that secretion of IFN-γ, which is also secreted by T cells in response to IL-12, was comparable between aged and young subjects.

### Increased differentiation to T follicular helper cells (Tfh) in aged as compared to young subjects

**Figure 4:**
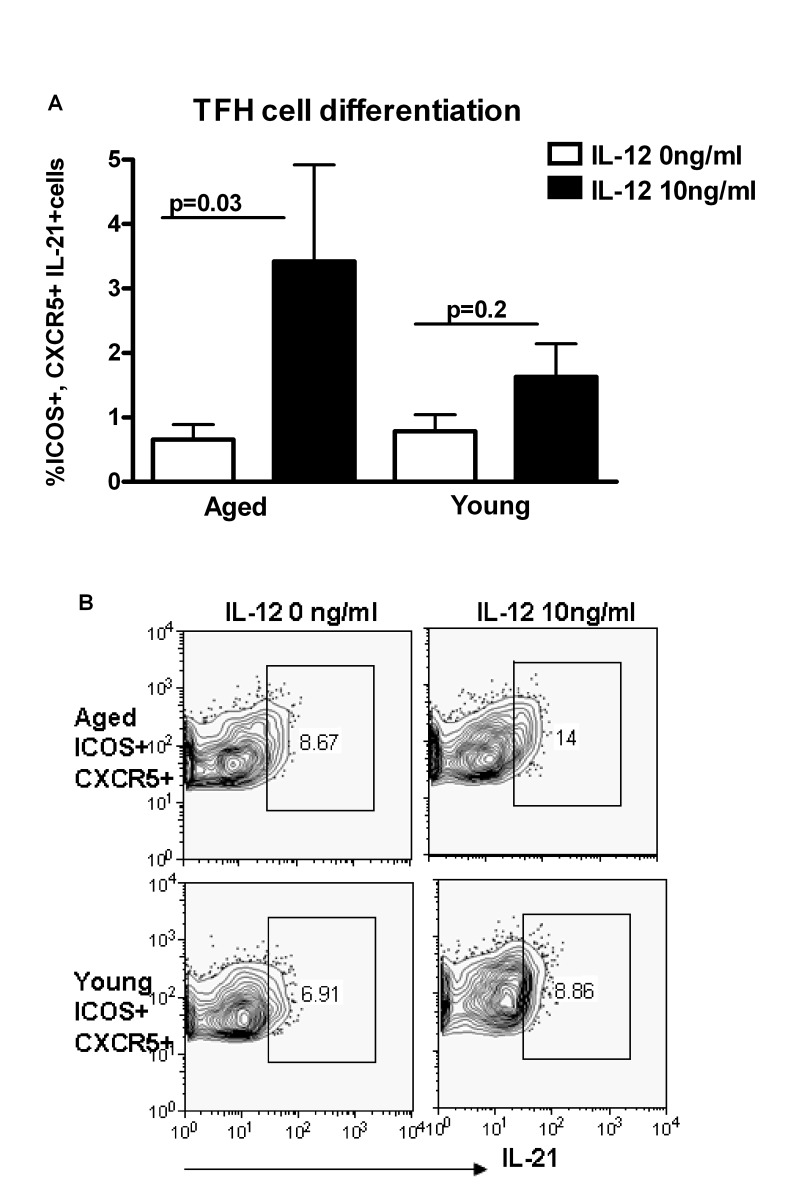
Increased differentiation to T follicular helper cells (Tfh) in aging subjects **A.** Bar diagram depicts the percentage of ICOS+, CXCR5+ IL-21+Tfh cells in aged and young CD4+ T cells after activation with IL-12 for 5 days. Data is mean +/− S.E. of 10 different aged and young subjects. **B.** Contour plot depicts the percent of ICOS+, CXCR5+ IL-21+ Tfh cells in aged and young CD4+ T cells after activation with IL-12 for 5 days. Data is representative of 10 such experiments.

IL-21 is secreted primarily by Tfh cells [12-17]. Therefore, we investigated whether there is increased differentiation of Th cells to Tfh cells in aging in response to IL-12. Tfh cells are ICOS+ and express the chemokine receptor CXCR5 which allows them to migrate to germinal centers and provide help to B cells [20]. Purified, naïve CD4+T cells from aged and young subjects were stimulated with ant-CD3 plus anti-CD28 antibodies and IL-12 as described in Figure 2. The percentages of ICOS+ CXCR5+ CD4+ cells secreting IL-21 were significantly higher (p<0.05) in aged subjects as compared to young subjects (Figure 4A & B). Therefore, IL-12 induces greater differentiation of aged CD4+ T cells towards IL-21 secreting Tfh cells as compared to young subjects. This suggests that signaling downstream of IL-12R may be altered with age.

### STAT-4 phosphorylation is altered in CD4+ T cells from aged subjects

**Figure 5:**
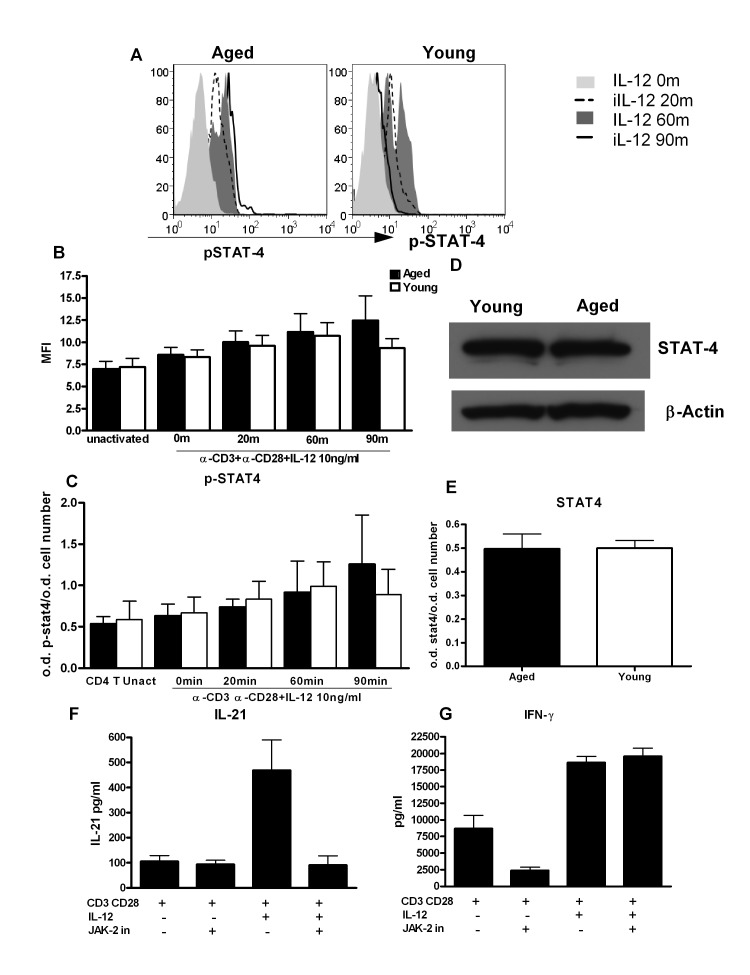
STAT-4 phosphorylation is altered in CD4+ T cells from aged subjects **A.** Histograms depict the phosphorylation of STAT-4 in naïve CD4+ T cells from aged and young at 0, 20, 60and 90 m after stimulation with IL-12. Graph is representative of 12 such experiments. **B.** Bar graph depicts the mean fluorescence intensity (MFI) of phosphorylation of STAT-4 in aged and young CD4+ T cells before and after activation with IL-12. Data is mean +/− S.E. of 12 different aged and young subjects. **C.** Bar graph also depicts phospho STAT-4 levels, the same as B using in cell ELISA. **D.** Western blot represents the level of non-phosphorylated STAT-4 in aged and young CD4+ T cells. Data represents pooled samples from 5 different aged and young subjects. **E.** Bar graph also depicts total STAT as in D using in cell ELISA. **F.** Graph depicts the level of IL-21 after treatment with JAK-2 inhibitor in aged CD4+ T cells. **G.** Graph depicts the level of IFN-γ after treatment with JAK-2 inhibitor in aged CD4+ T cells. Data is mean +/− S.E. of 6 different aged subjects.

Next we investigated whether signaling pathways responsible for IL-21 secretion in CD4+ T cells are altered with age. IL-12 mediates its biological effects by signaling through IL-12 receptors, which activate the Jak-signal transducer and activator of transcription (STAT) pathway [21, 22]. Among the STATs that are involved in IL-12 signaling, STAT-4 plays a critical role in IL-21 secretion particularly in humans [12, 23]. Therefore, we examined whether the age-associated alteration in STAT-4 signaling was responsible for the observed increase in IL-21 production. STAT-4 activity in aged and young PBMCs stimulated with IL-12 was determined by flow cytometry. Analysis was performed on gated CD4+ CD45RA+ naïve CD4+ T cells. As evident from Figure 5A, activation and kinetics of STAT-4 phosphorylation is significantly altered between aged and young naïve CD4+ T cells. In the young CD4+ T cells, phosphorylation of STAT-4 is observed at 20min, peaks at 60min and then starts to decrease. In CD4+ T cells from aged, the phosphorylation of STAT-4 also starts at 20min post stimulation with IL-12 but did not decrease over time and sustained at 90min when it has already downregulated in naïve T cells from young subjects. These results were further confirmed by in cell STAT-4 ELISA where a sustained STAT-4 phosphorylation was observed in aged CD4+ T cells (Figure 5C). Total STAT-4 levels were comparable in aged and young CD4+ T cells as determined by in cell ELISA and Western Blot (Figure 5D and E). Therefore, prolonged activation of STAT-4 may be responsible for the increased IL-21 secretion in aged CD4+ T cells.

To further confirm our findings, we utilized a specific JAK-2 inhibitor (1,2,3,4,5,6-Hexabromocyclohexane) which would inhibit phosphorylation of STAT-4 since it is downstream of JAK-2. Initial studies confirmed that this inhibitor was able to inhibit STAT-4 phosphorylation (supplementary Figure 2). Next, we determined the effect of JAK-2 inhibitor on IL-21 secretion. Purified CD4+ T cells were activated as described in Figure 2 except that JAK-2 inhibitor (JAK-2 inhibitor II) was added during activation with IL-12 to prevent activation of STAT-4. Five days later supernatants were collected and assayed for IL-21 and IFN-γ. Optimal concentration of JAK-2 inhibitor was determined and was found to be 50μM (data not shown). Addition of JAK-2 inhibitor resulted in more than 90% inhibition of IL-21 secretion (Figure 5F). In contrast, no inhibition of IFN-γ secretion was observed (Figure 5G) suggesting that prolonged STAT-4 activation plays a role in the induction of IL-21.

### Increased IL-21 secretion in aged subjects correlates with Cytomegalovirus (CMV) seropositivity

**Figure 6:**
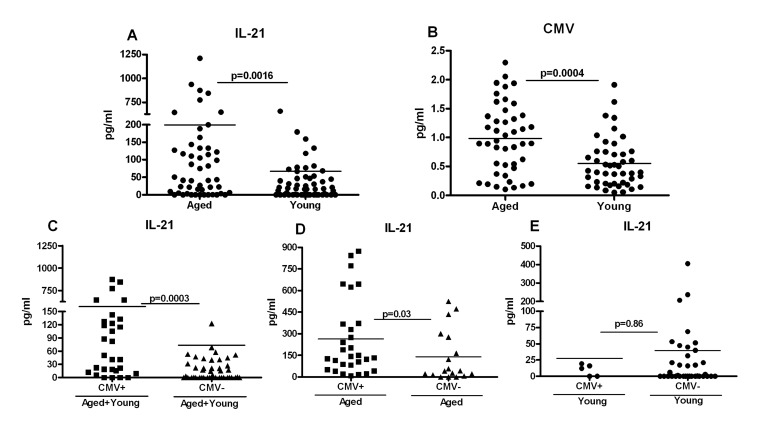
Increased IL-21 secretion in aged subjects correlates with Cytomegalovirus (CMV) seropositivity **A.** Dot blot depicts the level of IL-21 in aged and young plasma. Data is mean of 60 aged and 60 young subjects. Each dot represents one different subject. **B.** Dot blot depicts the level of CMV seropositivity in aged and young plasma. Data is mean of 50 aged and 50 young subjects. Each dot represents one different subject. **C.** Dot blot depicts the level of IL-21 in plasma of CMV+ and CMV- individuals. Data is mean of 38 CMV+ and 48 CMV- subjects. Each dot represents one different subject. **D.** Dot blot depicts the level of IL-21 in plasma of CMV+ and CMV- aged individuals. Data is mean of 32 CMV+ and 18 CMV- aged subjects. Each dot represents one different subject. **E.** Dot blot depicts the level of IL-21 in plasma of CMV+ and CMV- young individuals. Data is mean of 6 CMV+ and 30 CMV- young subjects. Each dot represents one different subject.

Next we investigated the mechanisms that are responsible for increased differentiation of aged CD4 T cells towards Tfh cells, IL-21 secretion and subsequent altered signaling. Recent studies have indicated that chronic viral infections increase naïve CD4 T cells’ differentiation toward Tfh cells, which are the major producers of IL-21 [24-27]. Aging is characterized by chronic viral infections such as CMV [28-31]. More than 90% of subjects over the age of 80 are seropositive for CMV antibodies. Therefore, increased Tfh differentiation and IL-21 production in aging may be a consequence of latent CMV infection. To investigate this, first we compared the percentage of Tfh cells (CD4+, ICOS+, CXCR5+) cells in the PBMCs from aged and young subjects (supplementary Figure 3). Tfh cell percentages were low but comparable between aged and young subjects. To further confirm our observations and to rule out the possible migration of Tfh cells to lymphoid organs, we compared the amount of IL-21 in the plasma from aged and young subjects. As is evident in Figure 6A, IL-21 levels were significantly elevated in plasma from aged subjects as compared to young subjects. Next, we determined the anti-CMV antibody index in aged and young cohorts. The number of CMV positive aged subjects was also significantly higher than young subjects (Figure 6B) which is in agreement with other reports. Further analysis revealed that IL-21 levels were significantly higher in CMV positive individuals (Figure 6C). Significant correlation between IL-21 production and CMV seropositivity existed in aged subjects (Figure 6D) but not in young subjects (Figure 6E). These data suggest that both chronic CMV infections as well as advancing age may be responsible for increased IL-21 secretion observed in aged subjects.

## DISCUSSION

Our study investigates the induction and differentiation of IL-21 producing Tfh cells in aged subjects. We demonstrate that CD4+ T cells from aged subjects display enhanced differentiation towards IL-21 producing Tfh cells, which is dependent on age-associated altered STAT-4 phosphorylation. Furthermore, we demonstrate that seropositivity to CMV correlates with the age-associated changes in CD4+T cells.

In contrast to mice, where IL-6 and IL-21 have been reported to be critical for generation of IL-21 producing Tfh cells, in humans, IL-12 produced by activated DCs was required to induce naïve CD4+ T cell differentiation towards Tfh like IL-21 producing cells [12]. Our findings suggest that IL-12p70 secretion in response to anti-CD40 and LPS is comparable between aged and young DCs (Figure 1A). Panda et al [7] have reported decreased secretion of IL-12p40 from aged human DCs in circulation after stimulation with LPS. One possibility for the discrepancy is that IL-12p40 is the common subunit shared by both IL-12p70 and IL-23 and does not represent the active form of IL-12.

Aged CD4 T cells produced increased amounts of IL-21 on stimulation with both aged and young DCs (1C, F,) suggesting that age-associated intrinsic changes in T cells are responsible for the increased IL-21 production. The difference was more apparent in anti-CD40 stimulated group. This is in agreement with previous findings where stimulation of DCs with anti-CD40 was found to be more efficient in inducing IL-21 from T cells. IL-21 was also not being produced by Th17 cells since IL-17 was found to be below the detection limits in the assay, which is not surprising since both anti-CD40 and LPS do not favor IL-17 production. LPS favors the generation of Th1 producing IFN-γ cells [32] which was increased in LPS -stimulated groups. The increased basal level of T cell proliferation and IFN-γ production in aged DCs plus aged T cell co-cultures (Figure 1B) could be due to an increased basal level activation of aged DCs. We have previously reported that aged DCs express increased basal level activation of NFκB [6]. The data from exposure of aged and young purified CD4+ T cells to IL-12 further confirmed that intrinsic changes in aged CD4 T cells are responsible for the increased IL-21 production by aged CD4 T cells (Figure 2).

Our data demonstrates that the increased IL-21 production by aged CD4+T cells is due to an increase in differentiation towards IL-21 producing Tfh cells (Figure 4A & B). More importantly, IL-21 increase correlates positively with CMV seropositivity (Figure 6). It has been recently shown that IL-21 production is increased during chronic viral infections and is essential in containing the infection via its action on CD8+ T cells [24-27]. Furthermore, the increased IL-21 production during chronic viral infection is due to increased differentiation of CD4+ T cells towards Tfh phenotype because of prolonged antigenic stimulation during viral persistence [24-27]. The increased IL-21 production by CD4+ T cells from aged may therefore be a consequence of chronic antigenic stimulation by CMV in the aged subjects.

Our observations in Figure 5 demonstrate that STAT-4 phosphorylation in response to IL-12 is sustained for a longer duration in aged CD4+T cells compared to young CD4+T cells. IL-12 in CD4+ T cells signals primarily through STAT-4. It has been reported that both IL-21 and IFN-γ are induced via the JAK2/STAT4 pathway [21, 22, 33]. Schmidt et al [12] have shown that STAT-4 is the primary regulator of IL-21 production in humans which is in contrast to mice where it is STAT-3 dependent. Wei et al [23] in CHIP-CHIP experiments also found that the IL-21 gene was a very prominent STAT4-bound gene whose epigenetic regulation was strongly STAT4-dependent. It may be that chronic viral infections such as CMV modify the response of naïve CD4 T cells to IL-12 inducing IL-21 secretion.

Increased IL-21 production by aged T cells may significantly impact immune functions in aging. Increased IL-21 production has been reported to enhance autoantibody production in autoimmune diseases because of its capacity to induce the differentiation of B cells towards antibody secreting plasma cells [34]. IL-21 also promotes the generation of IL-17 producing CD4+ T cells which are implicated in the pathogenesis of autoimmune and inflammatory disorders [35]. Recent studies suggest that increased production of IL-21 may be detrimental in fighting certain infections. Using IL-21−/− mice, Spolski et al. [36] demonstrated that IL-21 promotes the pathogenic inflammatory effect of Pneumovirus which resembles respiratory syncytial virus infection in humans. Aged subjects display increased susceptibility as well as severity to RSV infections. Increased IL-21 production by CD4 T cells during aging is another example of cellular hyper-function and hyper-activation which is considered as a basis of aging process. In theory rapamycin treatment should be able to reduce the IL-21 production in since it is considered effective in reducing the overstimulation of cellular signaling pathways [38].

In summary, we have demonstrated that IL-21 secretion from CD4+T cells is increased with age. Age-associated intrinsic changes in aged CD4 T cells and not increased IL-12 secretion by aged DCs is responsible for the enhanced IL-21 production. Increased IL-21 is a consequence of increased differentiation of Th cells towards ICOS+, CXCR5+ IL-21 secreting Tfh cells. Increased IL-21 production with age is due altered response of aged CD4 T cells to IL-12 which leads to prolonged STAT-4 activation. Finally, we also demonstrate that increased IL-21 produced in aged individuals correlates with CMV seropositivity. The consequences of the increased IL-21 production on the functions of immune system cells remain to be determined.

## MATERIALS AND METHODS

### Blood donors

Blood was collected from healthy aged (65-90 yrs) and young (20-35 yrs) donors. Aged subjects were of middle-class socio-economic status, and were living independently. Cohort description is provided in Table I. The Institutional Review Board of the University of California, Irvine, approved this study.

### Dendritic cells activation and culture with T cells

DCs were prepared essentially as described [37]. Briefly, purified monocytes from the aged and young PBMCs were cultured in the presence of 50 ng/ml human rGM-CSF (PeproTech, Rocky Hill, NJ), and 10 ng/ml human rIL-4 (Peprotech) for 6 days. The purity of the DC obtained was >95% as determined by the expression of CD14, CD11c and HLA-DR. DCs were stimulated with E.coli LPS (100ng/ml, Invivogen, San Diego, CA) and anti CD4+0 antibody (10μg/ml, R & D systems, Minneapolis, MI) for 24h. After overnight stimulation, supernatants were collected and stored at −20°C for IL-12p70 estimation using specific ELISA (BD Bioscience, San Jose, CA). Stimulated DCs were cultured with purified, CFSE-labeled allogeneic T cells from aged and young donors at a ratio of 1:10. Six days after culture, cells were collected and proliferation of gated CD4+ T cells was determined by measuring the dilution of CFSE dye. Supernatant collected was assayed for secretion of IL-21, IFN-γ and IL-17 by specific ELISA.

### CD4+ T cell culture

CD4+ T cells were purified from aged and young PBMCs by negative selection using magnetic beads (Stem cell Separation, Vancouver). Memory CD4+ T cells were positively selected using CD45RO+ magnetic beads; remaining cells (CD4+, CD45RA+) were considered naïve T cells. The purity was routinely found to be greater than 90%. Purified naïve and memory CD4+ T cells from aged and young subjects were stimulated with anti-CD3 and anti-CD28 tagged magnetic beads (Invitrogen, Carlsbad, CA) in the presence or absence of IL-12 (Peprotech, Rocky Hill, NJ) at concentrations ranging from 1ng-10ng/ml. Five days later the supernatants were collected and assayed for the presence of T cell cytokines, IFN-γ and IL-21 by specific ELISA (BD Bioscience, San Jose, CA).

JAK-2 inhibitor II (1,2,3,4,5,6-Hexabromocyclohexane) was purchased from (EMD Biosciences, Gibbstown, NJ) and used at a concentration of 50uM. This inhibitor prevents the activation of JAK-2 and downstream STAT-4. The procedure for JAK-2 inhibitor experiments was essentially similar except that JAK-2 inhibitor was added 30min after the addition of anti-CD3 plus anti-CD28 tagged magnetic beads and IL-12.

### Intracellular Cytokine Staining

Purified naïve and memory CD4+ T cells from aged and young subjects were stimulated with anti-CD3 and anti-CD28 tagged magnetic beads (Invitrogen) in the presence or absence of IL-12 as described above. On day 5 of the culture, cells were activated with PMA and ionomycin for 6h. Brefeldin A was added in the last 4 hr. Cells were collected and stained for surface CD4+, ICOS+, CXCR5+ (for Tfh cells) and CD4+, CD45RA+, CCR7+ (for naïve CD4 T cells) using directly conjugated specific antibodies. Subsequently the cells were permeabilized and stained for intracellular IL-21 using specific antibodies (BD Biosciences) and isotype controls.

### phospho-STAT-4 staining and cell ELISA

PBMCs from aged and young donors were stimulated with antiCD3, antiCD28 and IL-12 (10ng/ml) for time periods ranging from 0-90min at 37°C. Cells were then fixed with BD Phosflow fix buffer I for 10 min at RT, and subsequently incubated with CD4+ and CD4+5RA antibodies for 20 min at RT to stain for surface markers. After washing, cells were permeabilized with phosflow perm buffer III (BD Biosciences) and stained with phospho STAT-4 Alexa488 (BD Biosciences) for 30 min at RT. Mouse IgG1-Alexa 488 (BD Biosciences) was used as isotype control. Stained cells were acquired on FACsCalibur and gated CD4+ plus CD45RA+ double-positive naïve CD4+ T were analyzed for phospho STAT-4 using Flow jo.

STAT-4 activity in aged and young CD4+ T cells was determined by FACE in-cell ELISA kit (Active Motif, Carlsbad, CA). The experiment was performed as per the manufacturers’ protocol. Briefly, 2×105/well of purified CD4+ T cells from aged or young were added to poly-l-Lysine coated 96 well culture plates. The cells were allowed to adhere for 1h. Subsequently, non-adherent cells were removed and remaining adherent cells were activated with anti-CD3 plus anti-CD28 containing beads, in the presence or absence of IL-21. Activation was performed for a duration ranging from 20min to 90min. Cells were then fixed with 8% formaldehyde. Subsequently, phosphorylated and total STAT-4 levels were quantified using specific antibodies in an ELISA reaction. The absorbance was read at 450nm. After reading, the wells were washed and stained with crystal violet to quantify the number of cells. Crystal Violet is an intense stain that binds to the cell nuclei and gives an OD 595nm reading that is proportional to cell number. The STAT absorbance was normalized to the number of cells.

### CMV ELISA

Commercially available CMV IgG specific ELISA kits (Cortez Diagnostics Inc, CA) were used to determine the CMV seropositivity in plasma samples from aged and young subjects. Manufacturer's instruction was followed and CMV antibody index was determined.

### Statistical analysis

Statistical analysis was performed by in house statistician. Within group differences between unstimulated and stimulated conditions were tested using paired t-tests. Mann Whitney or Wilcoxon signed rank tests were used to measure significance between aged and young groups. Values of p < 0.05 were considered significant.

## SUPPLEMENTARY FIGURES


