# Supplementary material for: Increased IL-21 secretion by aged CD4+T cells is associated with prolonged STAT-4 activation and CMV seropositivity
Source: Aging (Albany NY). 2012 Sep 29;4(9):648–59. doi: 10.18632/aging.100490 (PMC3492228; doi:10.18632/aging.100490)
Supplement: Supplementary file 1 [file aging-04-648-s001.pdf]

36. Spolski R, Wang L, Wan CK, Bonville CA, Domachowske JB, Kim HP, Yu Z, Leonard WJ. IL-21 Promotes the Pathologic Immune Response to Pneumovirus Infection. J Immunol. 2012 Jan 11.

37. Agrawal A, Agrawal S, Cao JN, Su H, Osann K, Gupta S. Altered innate immune functioning of dendritic cells in elderly humans: a role of phosphoinositide 3-kinase-signaling pathway. J Immunol.2007; 178: 6912-6922.

38. Blagosklonny MV. Rapamycin and quasi-programmed aging: four years later. Cell Cycle. 2010;9:1859-1862.

SUPPLEMENTARY FIGURES

Supplementary figure 1

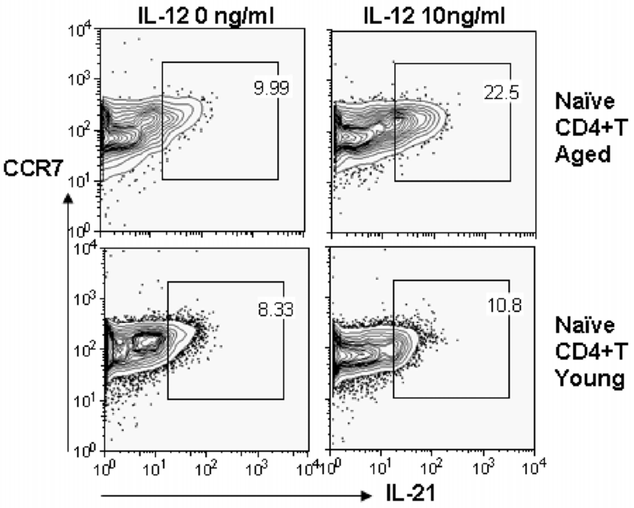

Purified CD4+ T cells from aged and young subjects were stimulated with anti-CD3+anti-CD28 containing magnetic beads +/- IL-12 for 5 days. Intracellular staining for IL-21 was perfomed (see methods). Gated CD4+ CD45RA+ CCR7+ naive T cells were analyzed for the presence of IL-21 by flow cyometry. Data is representative of 6 such experiments.

Supplementary figure 2

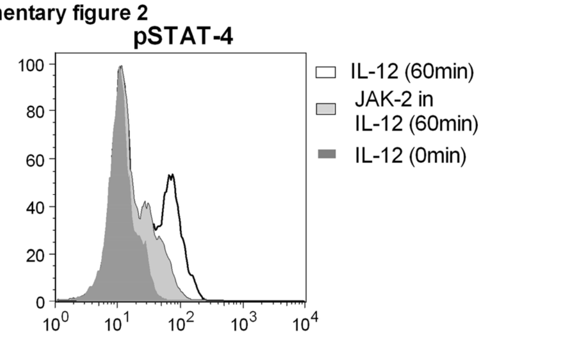

Histogram depicts the phosphorylation of STAT-4 in naïve CD4+T cells +/- JAK-2 inhibitor after stimulation with IL-12 for 60min.

Supplementary Figure 3

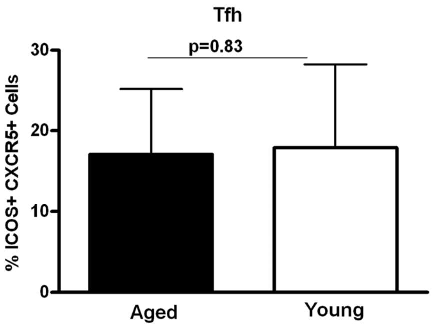

PBMCs from aged and young subjects were stained for the expression of CD4, ICOS and CXCR5 using specific antibodies. Bar graphrs depict the percentage of ICOS+ CXCR+ CD4+ cells in the PBMCs of aged and young subjects. Data is mean +/- S.E. of 12 subjects.
